# Supplementary figures and images for: Association of a rapidly selected 4.3kb transposon-containing structural variation with a P450-based resistance to pyrethroids in the African malaria vector Anopheles funestus
Source: PLoS Genet. 2024 Jul 29;20(7):e1011344. doi: 10.1371/journal.pgen.1011344 (PMC11309504; doi:10.1371/journal.pgen.1011344)

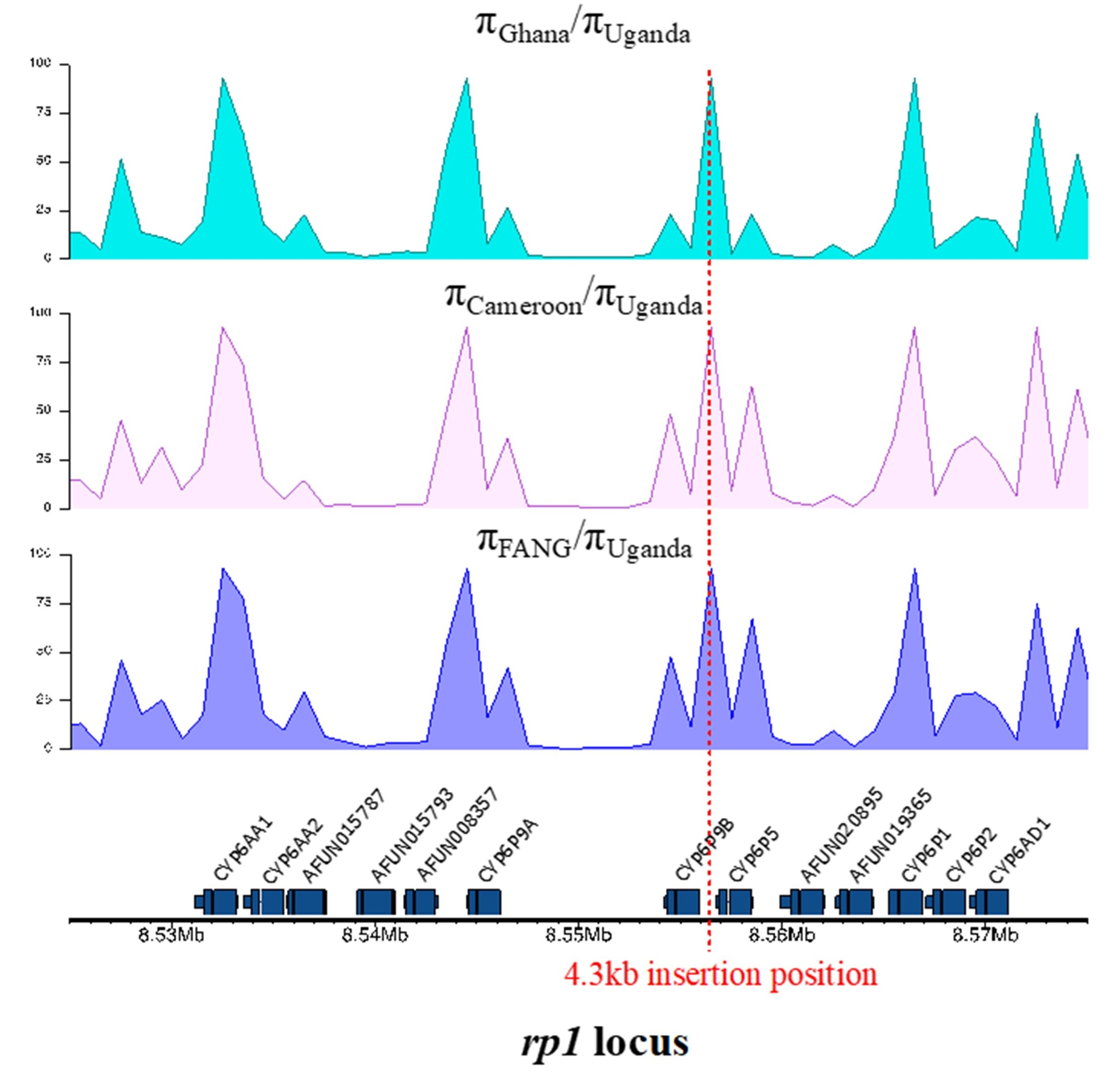

Supplement: S1 Fig — A peak indicates a loss of diversity in Uganda versus the comparator population. The SV insertion position is labelled with the red-dashed line and gene positions are indicated below the plots, genes are labelled by their cytochrome P450s name or there VectorBase identifier if not a P450 gene. An upper-bound of 100 was placed on peaks to ensure interpretable plots, these represent windows with very little to zero diversity in Uganda. (TIFF) [file pgen.1011344.s001.tiff]

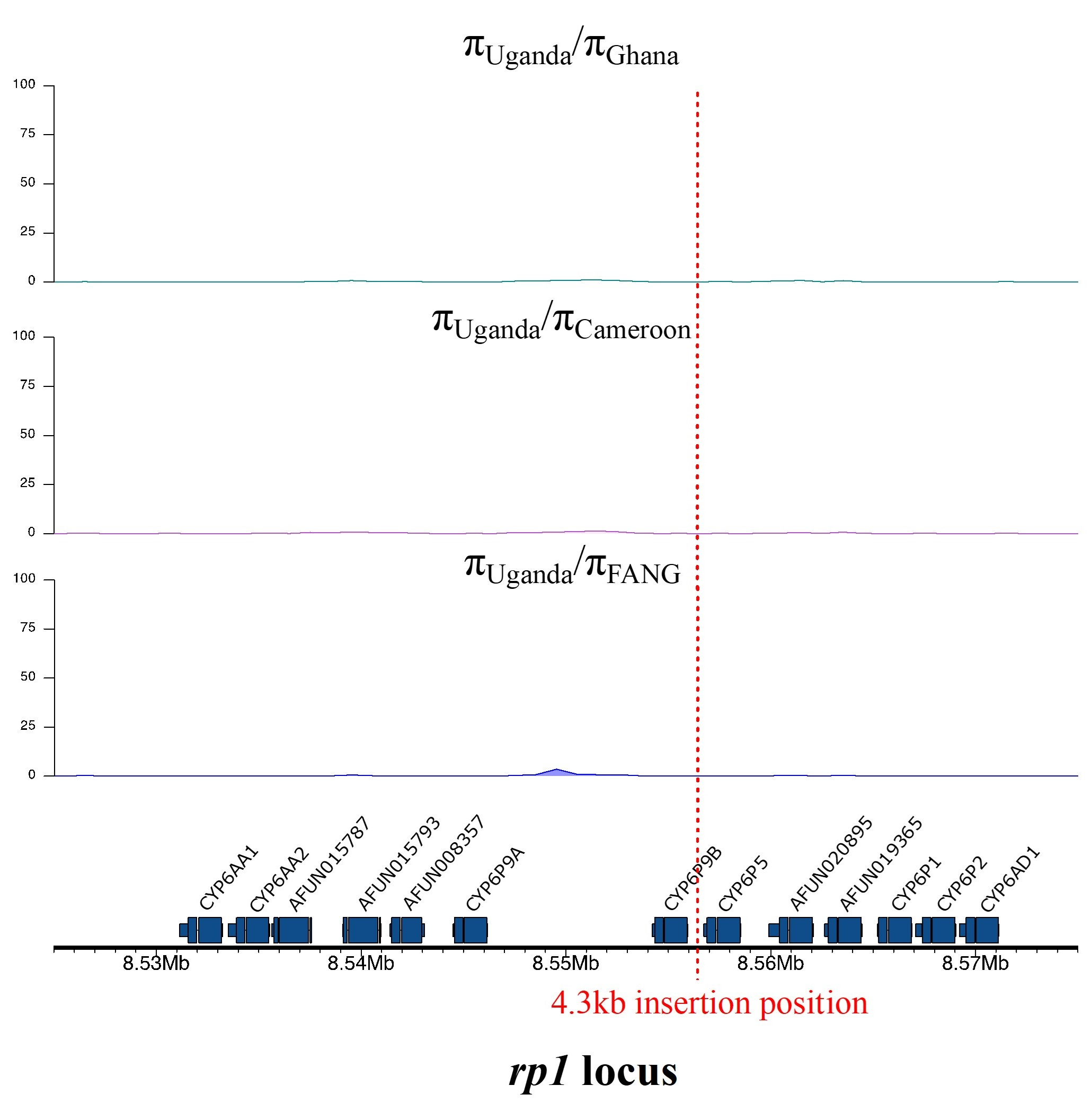

Supplement: S2 Fig — This is the reverse of S1 Fig in the main text as Ugandan diversity is divided by comparator populations. It shows no peaks indicative of a drop of nucleotide diversity in the denominator population unlike the peaks shown for Uganda in S1 Fig. (TIFF) [file pgen.1011344.s002.tiff]

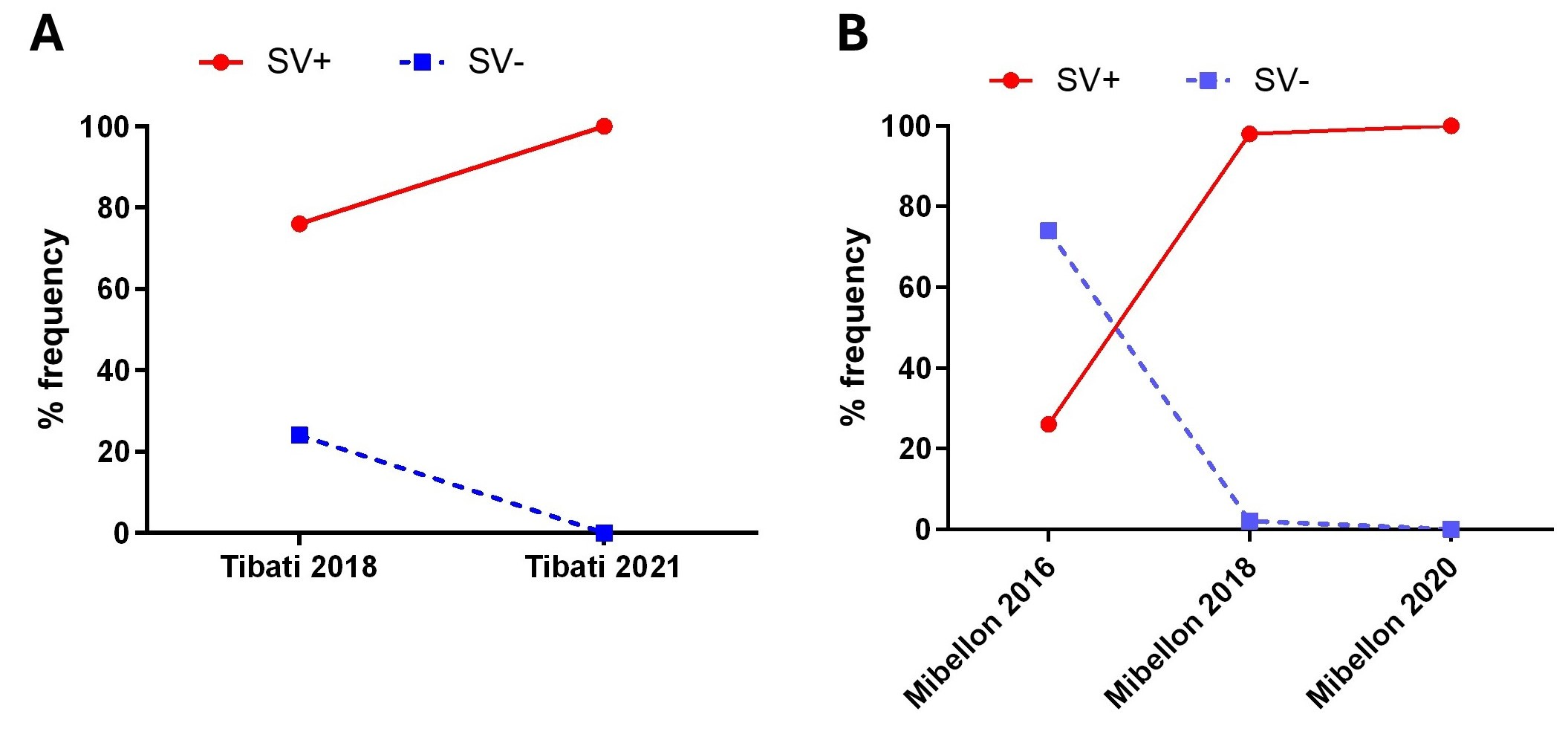

Supplement: S3 Fig — (A) Allelic frequencies of 4.3kb SV in Tibati showing a decrease in SV- allele and increase in SV+ over the time. (B) Allelic frequencies of 4.3kb SV in Mibellon showing a decrease in SV- allele and increase in SV+ over the time. (TIFF) [file pgen.1011344.s003.tiff]

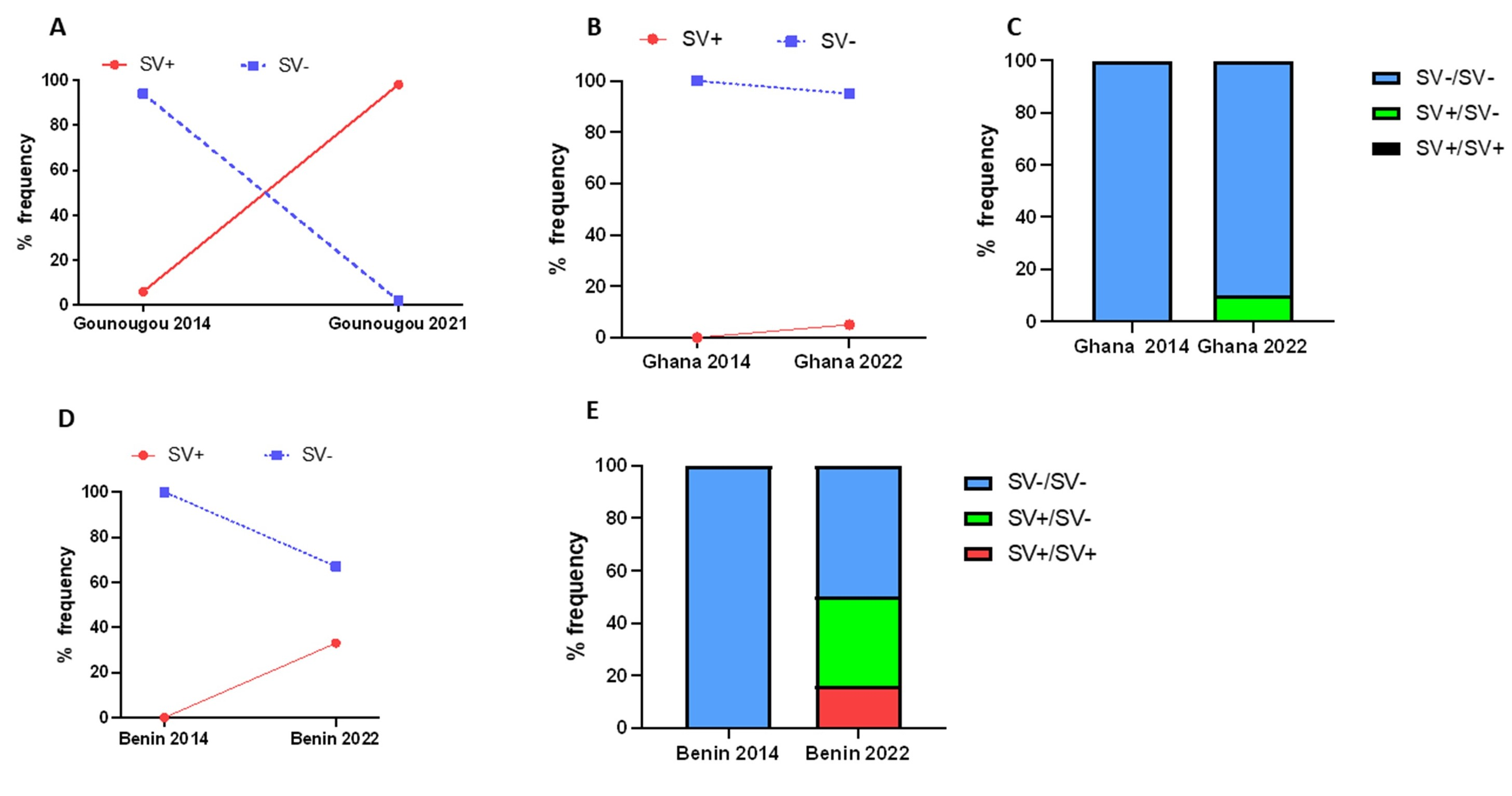

Supplement: S4 Fig — Changes in frequencies of the 4.3kb SV over time in (A) Gounougou, (B and C) (D and E) Benin and Ghana. (TIFF) [file pgen.1011344.s004.tiff]

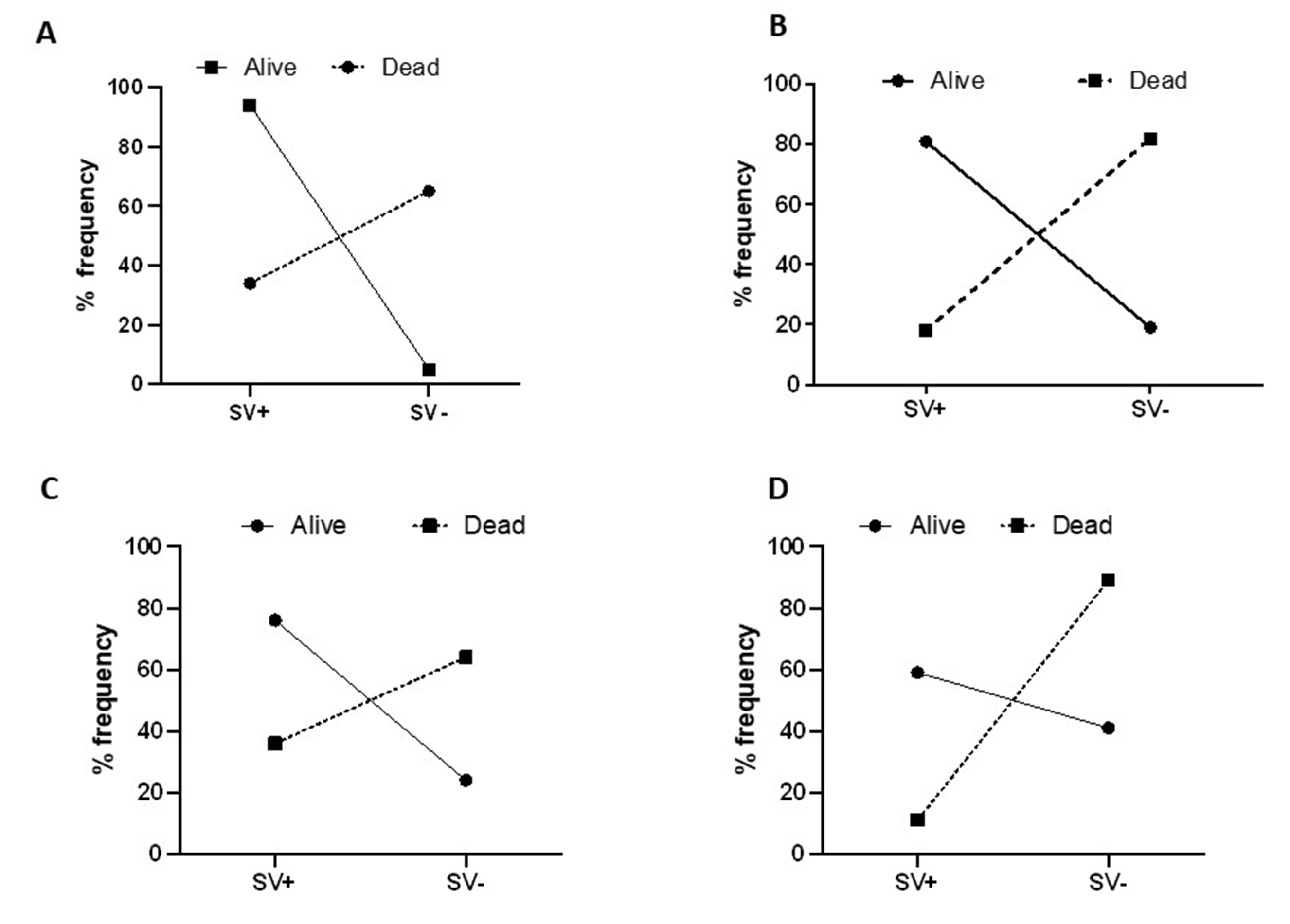

Supplement: S5 Fig — (A) Genotyping results of the 4.3kb SV among the Gounougou 2018 alive and dead deltamethrin post exposure reveal a strong association between the 4.3kb SV and ability to survive. B) illustrate the strong association between 4.3kb SV and the ability to survive exposure to deltamethrin by looking at its genotypic and allelic distribution among dead and alive F3 Elende-Fang crossing mosquitoes. C) illustrate the strong association between 4.3kb SV and the ability to survive exposure to Permethrin by looking at its genotypic and allelic distribution among dead and alive F3 mibellon-Fang crossing mosquitoes. D) illustrate the strong association between 4.3kb SV and the ability to survive exposure to α-cypermethrin by looking at its genotypic and allelic distribution among dead and alive F3 mibellon-Fang crossing mosquitoes. (TIFF) [file pgen.1011344.s005.tiff]
