# Supplementary material for: Association of a rapidly selected 4.3kb transposon-containing structural variation with a P450-based resistance to pyrethroids in the African malaria vector Anopheles funestus
Source: PLoS Genet. 2024 Jul 29;20(7):e1011344. doi: 10.1371/journal.pgen.1011344 (PMC11309504; doi:10.1371/journal.pgen.1011344)
Supplement: S1 Text — (DOCX) [file pgen.1011344.s006.docx]

**5. The 4.3kb SV is associated with pyrethroid resistance**

We next aimed to establish if this 4.3kb contributes to pyrethroid resistance, thus explaining its swift increase in frequency in field populations of *An. funestus* from Central and East Africa.

**5.1 Genotyping of field samples reveals a correlation between the 4.3kb SV and pyrethroid resistance.**

Gounougou samples obtained from the deltamethrin 0.05% WHO tube bioassays in 2018 (when the 4.3kb structural variant was not yet fixed) were genotyped to determine any association between the 4.3kb SV and deltamethrin resistance phenotype. The mortality rate for deltamethrin was 49.8±11.6% with a knockdown rate of 94.5%. Genotyping of 77 mosquitoes exposed to deltamethrin (35 alive and 42 dead) revealed a strong association (χ2=33.8; P<0.00001) between this structural variant and resistance to deltamethrin. Within the homozygous (SV+/SV+) genotype, 74.4% (32/44) of individuals were found to be alive, while 25.2% (11/43) were deceased (Figure 3A; Table 1). Among those with the heterozygous genotype (SV+/SV-), 22.2% (2/9) were alive, contrasting with 77.8% (7/9) that were not alive. Notably, the majority of individuals lacking the structural variant (SV-/SV-) exhibited a mortality rate of 96% (24/25), with only 4% (1/25) surviving (Figure 3A; Table 1).

The odds ratio analysis revealed a robust positive association between the 4.3kb SV genotype and survival following deltamethrin exposure. Individuals with the SV+/SV+ genotype demonstrated a significantly higher likelihood of survival compared to both SV+/SV- [OR: 10.2; CI: 1.8-56.5; P = 0.0080] and SV-/SV- [OR: 69.8; CI: 8.4-578.5; P = 0.0001]. The elevated odds ratio (10.18) for SV+/SV+ versus SV+/SV- suggests an additive effect of each 4.3 kb structural variant allele. Allelic frequencies showed that 94.3% of survivors possessed the SV+ allele, contrasting with the 5.7% lacking the 4.3kb SV allele, emphasizing a robust association with deltamethrin resistance (OR: 8.4; CI: 8.4-41.7; P < 0.0001). Among deceased mosquitoes, 65.5% lacked the 4.3kb SV (SV-), while 34.5% had the 4.3kb SV (SV+), indicating reduced mortality likelihood in individuals harboring the 4.3kb SV compared to those without (Figure S5; Table 1). The survival probability increases with two copies of SV+ copies compared to just having one (refer to Table 1 for details).

**5.2 The 4.3kb SV is reducing the efficacy of LLINs through genotyping of cone assays samples.**

To validate the ability of the 4.3 kb SV marker to predict the impact of resistance on the efficacy of LLINs, we genotyped F_1_ Gounougou individuals obtained from cone assay with pyrethroid-only and PBO-based nets. A mortality of 100% was obtained when the Kisumu susceptible strain was exposed to these nets. Genotyping of dead and alive mosquitoes from Gounougou 2018 showed a differential survival for mosquitoes with the 4.3kb SV depending on the bed net used. Mosquitoes were phenotypically resistant to almost all the nets tested with mortality rates below 80%, 9±3.8% for Olyset, 53±11.5% for Olyset plus, 15±3.0% for PermaNet 2.0. PermaNet 3.0 was the most effective, with mortality rates of 100% when a piece of the top was used while the side gave 33±5.6%. The knockdown rates were 39±6.4% for Olyset, 30.1±7.5% for PermaNet 2.0, 72.9±24.4% for Olyset plus and 100% for PermaNet 3.0.

A significant difference in the distribution of genotypes of the 4.3kb SV was observed for PermaNet 2.0 between dead and alive mosquitoes (χ^2^= 112.7; P< 0.00001) (Figure 3B; Table 1). No mortality was observed for mosquitoes with either 1 (SV+/SV-) or 2 (SV+/SV+) alleles for the structural variant. The alive PermaNet 2.0 individuals were composed of 74.1% (17/27) SV+/SV+, 22.2% (6/27) SV+/SV- and 14.8% (4/27) SV-/SV- while all the dead were SV-/SV- (100%) (Figure 3B; Table 1). In comparison, SV+/SV+ homozygote mosquitoes and heterozygote (SV+/SV-) mosquitoes exhibited a significantly higher likelihood of survival following PermaNet 2.0 exposure when contrasted with mosquitoes entirely lacking the 4.3kb SV (SV-/SV-) (OR: 201; CI: 12.15-3325.23; P = 0.0002). This Suggests an association between harboring this structural variant and the capacity to survive exposure to the PermaNet 2.0 bednet, whether in the homozygous or heterozygous state. Individuals possessing a single 4.3kb SV+ allele exhibit a significantly enhanced ability to withstand exposure to PermaNet 2.0 compared to those with the SV- allele, as indicated by a high odds ratio of 82.93 (CI: 4.98 - 1379.74, P = 0.0021) at the allelic level (S3A Fig.; Table 1).

Genotyping of 43 individuals (with 40 alive and 3 dead) exposed to Olyset, impregnated with permethrin, was inconclusive as only 3 mosquitoes were dead. Analysis of the Olyset plus exposed mosquitoes revealed a significant difference in the survival ability of 4.3kb SV genotypes (χ^2^= 56.4; P< 0.00001). The distribution of the 4.3kb SV in the alive individuals was 64.3% (9/14) for the SV+/SV+, 0% for the SV+/SV- and 35.7% (5/14) for the SV-/SV-. Among the dead individuals, we had 45.5% (10/22) SV+/SV+, 9.1% (2/22) SV+/SV- and 45.5% (10/22) SV-/SV-. There was a marginally significant differences in surviving exposure to Olyset Plus between the SV+/SV+ and the SV-/SV- genotype (OR: 1.8; CI: 1.0 to 3.3; P = 0.04). A similar result was obtained at the allelic level, with 64.3%of the alive having the SV+ allele and 35.7% having the SV- allele, while for the dead, both alleles were equally represented at 50% each (Table 1). A weak significant survival likelihood was observed when comparing SV+ and SV- (OR: 1.8; CI: 1.03 to 3.32; P = 0.04).

**5.3 Genetic crosses further reveal an association between the 4.3kb SV and pyrethroid resistance**

Another approach was to use samples generated from crossing Elende field mosquitoes (SV+/SV+) and FANG lab susceptible colony (SV-/SV-), allowing them to interbreed and later conduct bioassays.

Results from deltamethrin (0.05%) bioassay at F_3_ exposed for 60 minutes revealed 80.1 ± 5% mortality rates. Genotyping of the 4.3kb SV revealed a strong association between the presence of this structural variant and the ability to survive exposure to deltamethrin. SV+ was significantly associated with survival to deltamethrin exposure (OR: 19.4; CI: 9.5 to 39.6; P< 0.0001) (Figure 3C; Table 2). Homozygote SV+/SV+ mosquitoes had significantly higher likelihood of surviving than the heterozygotes SV+/SV- (OR: 37; CI: 2-689.9; P=0.016) and the homozygote lacking the SV SV-/SV- (OR: 1517; CI: 28.6-80383.1; P = 0.0003) genotypes. The heterozygotes also had higher chances of surviving than homozygotes SV-/SV- (OR: 41; CI: 2.2 to 761.8; P = 0.01).

Another crossing generated with the field Mibellon males (fixed for the SV+) crossed with FANG females (without this SV) at F_3_ revealed a similar association with permethrin (0.75%, type I) and alpha-cypermethrin (0.05%, type II). This SV was found to be linked with the ability to survive permethrin exposure (OR: 5.6; CI: 3.1 to 10.4; P< 0.0001) (Figure 3D; Table 2). At the genotypic level, the SV+/SV+ genotype had increased chances of being alive after exposure to permethrin compared to the SV+/SV- (OR: 6.4; CI: 2.0-20.8; P=0.0018) and the SV-/SV- (OR: 17.7; CI: 4.3-72.3; P = 0.0001) genotypes. Heterozygotes still had higher odds of surviving than the SV-/SV- (OR: 2.8; CI: 0.7 to 10.4; P = 0.0135) (Figure 3D; Table 2).

Furthermore, we examined the frequency of the 4.3kb SV in the alpha-cypermethrin 60 minutes alive and 10 minutes dead. Among the alive, 59.09% had the SV+ while 40.91% lacked the SV- (Figure 3E; Table 2), showing that this SV is also associated with survival to alpha-cypermethrin exposure (OR: 116.4; CI: 5.5 to 24.5; P< 0.0001). Calculating the odds of having the SV and surviving revealed a positive association between both. The SV+/SV+ genotype is associated with survival to alpha-cypermethrin more than the SV+/SV- (OR: 3.05; CI: 0.8-11.2; P=0.009) and the SV-/SV- (OR: 72.7; CI: 18.9-278.8; P < 0.0001) genotypes. The SV+/SV- genotype also had more chances of surviving compared to the SV-/SV- (OR: 24; CI: 10.5 to 54.2; P < 0.0001) (Figure 3E)

**4.3kb Structural variant sequence**

>4.3kb_SV

TGTTGAATTATCCCAATTGGTAAATTTAAAACATCAATTGATTATAAATTCCATTTATTATGTACATTTGGTAGTAGTAGTAAGTTTTATATATTTCGATTTTCAATTTCATTTTATAATACAAGGTTTAGTATTGTAACATTAGAACTTAAGCGGGTTCGACTATTGTGTCTTTCCACGCAAAATTTAACTGCTGATCTTGCGTTGCGGTTTTAACAGGATTTAAACTACCTTGAGGGCATTCTGAATTATGAGCGTGGGAAAAAATACGAGTAAAACCCACAACAAGTTCAAAAAAAAAAAAAAATCTTTTTACCTTACAAGCTATACTTATATCTATGCCTTATAATAATACTTATTTTAACTAACCTAGTAATGTTGACTTGATTGGCTGTATATTGTTTAGTTTGTAGTTTAATTCGTTAACTATTTCAAGAGGGTTGGATTGGCATTTTGATGTATATTTGTTTTGAATATTGGTTAGGTGTTCTTTTAGCAGTGGTGTGTTACATTTTTCGTGAAGTAAGGATGTGCGGTACCAAGGAGGAACATCTAAAATAGATTTTAGATATTTGTTTTGAATCCTTTGCAAAGTTAATTTATGGCATTTTGCGCAATCAATCCATGCTGGAGAGGCGTATGTTATAATTGGCCGTATTGCCACCTTATAAAGGAGTAATTTGTTTAGAAAATTTAGTTTTGATTTTCTGTGCAGGAAACTGTATAATATTTTAAACATTTTTTCACATTTTAGCAAGGTAGCTTCAGTGTTTGTTTTGAAGGTTAGTCTTTTATCTATTTGAATCCCTAAGTATTTGACCGCATTTTTCCATGGAATGTCGCTGTTGTTGTAGATCACATTTTTGTTTGGCAGTTTAGTAGCTTTTTTGAATTTGGTGAAAAATATTGCCTCAGATTTCGAACCATTGACTTTCAGCTTCCACTTGACACAGTACGCATTGTATTTTTTAAGTGCTACGTTAAGTGTTTTAATAACGGTTTTGGCATGCTTTGCTGAGGATGTTATAGCTATGTCGTCGGCGTATATGTATTTTGAGCAATTTTTCATTTGTGGTATATCTGAAGTATAGATATTGAACAGAAGAGGGGATAATGTACTGCCCTGAGGAACTCCGGCAGTGGGAGTGTAGGCTTCTGATTTGGTTTTACCAATGTGAATAAAGTTTTGTCTATTTTTAAGGAAGGATTCGATTATTTTGATAAGATGTGTTGGAAATTTAAAGTTGATTAATTTGTGTAATAGTCCCTGATGCCAAATTTTATCGAAAGCTTTTTCGCTGTCTAGAAGAACAAGACCTGTTGATTTTCCTTCTCGTCTGCTTTCCTTTATGTTTTTTGTCAATCTGTGAATTTGGTGCGTTGTGGATAAAGACGGTTGGAATCCGAATTGCTCGATTGGGATGATGTTATTGTTCGATGTGTGTGATCTAAGCTGAATCTCAATGCATTTTTCGAATAATTTACCTAGGCAACTTAAAAGGCTGATAGGTCTATAACTTTCTGGTTTGGAAGGATCTTTACCTGGTTTTGGAATTGCGATAACTTTAGCTATTTTCCAGGCACTGGGAAAGTAGCCAAGTTTAAGGCATCCATTAAATATGGTACTAAGATATACTATTGCTTTTCTTGGTAGTCTTTTCAGCGATCGATTATTTAGGTTGTCACTACCGGTGGATTTTTTATGCTTTAATTTTGACAAAAGTTTCTTAACATCTTTTGGTTTTACCATGTTATGAATGATTTGTTCTGTTTTTATAGATGTCTTTTGTAGTTTGCGAATGTCTTTGGCAACTTTCGCTTCCACTGTGCTGCTGGTGTTGTATGTGATTTTATGTGCTGTTTCAAAATGTTCCTTAAGTGTTTTACATTTTTCCTCGGAAGTGATTAAGGTTTTGTTCTCTATTTTCAATGGTGGGACTGATGTTGCGTTATTTTTAATAATTCGTACGAATTTCCAGAGCTGATTGTTATTTTTGAATTTATCGTTATTAATTTCTATTAATTTTTTACCCCACTCTCTATTTCGCAAGTCGGATAACGCTAGTTCAATTTTCTTTTTGAGCCGGTAGTACAACTGTTTTAGTGCAAGGTTTTGTCTGTTTCTTTGCCATCTCTTGCGGATAATATTTCGTATTTTTATGAGCGAGATTATTTGTGGAGGTATTATTAGTCTGTAAGTACTGGGAGTTGTGAGTGGAACTGCTAAGTTTTGAGCTGATATGATGGTTGTAGTGAGCTTTTTAATCATTTCGTCTATTTGTATATGGTTTTTTATGTCACTGTATTGTGTCTGCAATGGATTGATCGTCGTATCTATATGATCTTTAAATACCTTCCAGTTTGCTTTGCTGTAATTAAACACTTTTTGTTCGGGATTTTTTGTTGTTTTTGTGGTATAGACTGAAAAGTTTATGGGAAGGTGGTCTGAGGTCATGCAGGTTTTGGTGGATAAGTCTGATATTTCATACGTGATATTTGTTAAAGTTAAGTCTATTGTGGAGGGGTTTCGAGACGCGTCAGATGGATAGTATGTAGGTGAAATAGGATGATGTATTGTGAATTTGCCCGTTTGCATTTCTCTAAAAAGGCAATTTCCAGCTGAGTTTGTTGAGCAACAGTTCCATAGTCTGTGTCTGGCGTTAAAATCGCCACATATAAGAAAATTATCTTTCCTATTAGTTAAAGTTTGTATATCGCTTTTAAATTTCCCTACATTGATGTTTGCACCAGGATGGTATGCTGTGATAAATGTAATGGTTCCTTTTGTGGTGAATAATTGGATTTCTAATGATTCTATTATTTTAAGGTTTTGAACTTTTCGTACTTTGTGGTTAATGTGGTTTTTTATCAAAATAATGATTCCTCCTTTGGCACCGTCTATCCTGTCTAACCTGTGCGTTGTGTAGTTAGGTAATGAGAATTTTTGATTCGGCTTAAGAAAAGTTTCTACTATGCCAATCAAGTCAACATTATTATTATCTAGGTACTGTATTAGTTCATGTTTTTTGTTGGAGATGCCGTTTGCATTCCAGTAGGAAAATGAGATATTATTTGCCACCGTCATCACGGGAAGCAAAATTTCTGAACTATTTGAAAGACTACCCTAATTTGATCTTCCTTGGTTGAACACTTTCTTAGGCACGAAAACGTTTCAGTTATAATGTGGGCTAGTTCATCAGGCCCAAAGAGATCGTTCTGATTAGACGTAGTTACATTTGCATATGTGTTAGTTTGCGCACTATGCGTTCGCTTCGGATAAGTGTGGATGCTAGGGTTTTGTGGAGTCTGTGAACTCGAGGGTAAATGTGGGAAGTCTTGAAGAGATGTTGGTACCTGTTTTGGTTTTGTTTGTATGTGTTTGGTTCTATCTCTCTTGCTCTGCCGTTCCGCGCATCCGGAGAAATTTGCTGTGTGATTGCCACCACAGTTTGCACATTTTAGCTTGTGCGTTGGGATTACACCGTCCGTTGACCCTTTTGCTACTGGACACTCATTTGATGCGTGAAGATCTGCACACTTGATACATTTAGGTGGCTTGTAACAATTTGCCGCTCCGTGACCGAATCGTTGGCAATTTTTGCATTGCATTATACTGCTGCTGTTTGTATAATATCGCCAGTACACCGCCTGATGATCTAATGCTTTTATGGAACGCAGCATCTTCAAGTTAGCTGTGCCTTTGGTGAAGTGAAGCAAATATGTTGCTTGCTCGTCGTAGCGCTTTTTGGGTACTGCTAGCTTTTTTATCGCCACAGGTGTCAAACTTTCCTGTCTGAGGATGTTCTCGATTTGATCAATTGGCATGTCGATAAGCCCCATTAGAACAATTTTTGTTGTTCTATCTTCCTCCAGTTGATATGTGTGGAAGTTCGTGTTGATCTTTTGAAAGTGGCTCACCACTACCTTAAAATCGCTTACTGTAGTAGTGCGAACTACTATTCCTTTATTCGTAACAGATGTCGTATATCTTACGACACCAGCGGCGATAATTTTTTTGTGGATGTCTCCGATGTTGTTGCTATCAACCACTATTGGTGGTGGTGTTTTGGCTGTTGATGTTCGTTGCGTTGAGTTAGCATTATTGTTAACGATCTCCTGATCGTCTATTGTTTGAAGCAGTTCAAATTGGTTCGCTTGTTCCAACTGCCTTTTTGTTTTAGATTTGCAGCTTCCATCTACCGAGGATTGAAAAGCGGTGAGAGGCAAAGTTGCCCTCCTCTTGCTCACCATACGTTTAATGCGACCCATTTTGGGTGCACGAGAGAGAGAGAGACACTGAGATTTGCACTTTTTTATATCTGATTCTTGATTAGCTCTCGAGAAACACGTCAGCACGCATTCGAGTGTGGGAAGCTACTGA
