# Supplementary material for: Association of a rapidly selected 4.3kb transposon-containing structural variation with a P450-based resistance to pyrethroids in the African malaria vector Anopheles funestus
Source: PLoS Genet. 2024 Jul 29;20(7):e1011344. doi: 10.1371/journal.pgen.1011344 (PMC11309504; doi:10.1371/journal.pgen.1011344)
Supplement: S1 Table — (DOCX) [file pgen.1011344.s007.docx]

**S1 Table.** Additional sequencing primers were used to sequence the 5.5 kb intergenic sequence.

| Name | Sequence (5’-3’) |
| --- | --- |
| UGA_seq1 | CATTTATTATGTACATTTGGTAG |
| UGA_seq2 | TGGTTGATAGCAACAACATCG |
| UGA_seq3 | ACGGTTTTGGCATGCTTTGC |
| UGA_seq4 | ATGCAAACGGCATCTCCAAC |
