# Supplementary material for: Association of a rapidly selected 4.3kb transposon-containing structural variation with a P450-based resistance to pyrethroids in the African malaria vector Anopheles funestus
Source: PLoS Genet. 2024 Jul 29;20(7):e1011344. doi: 10.1371/journal.pgen.1011344 (PMC11309504; doi:10.1371/journal.pgen.1011344)
Supplement: S2 Table — (DOCX) [file pgen.1011344.s008.docx]

S2 Table. qRT-PCR Primers

| Gene | Forward primer | Reverse primer | Expected size (bp) |
| --- | --- | --- | --- |
| CYP6P9a | CAGCGCGTACACCAGATTGTGTAA | TCACAATTTTTCCACCTTCAAGTAATTACCCGC | 92 |
| CYP6P9b | CAGCGCGTACACCAGATTGTGTAA | TTACACCTTTTCTACCTTCAAGTAATTACCCGC | 97 |
| CYP6P5 | ACGTAATCAACGAAACGCTTCGTA | TCGGTATCTGCACGATTGTT | 126 |
