# Supplementary material for: Association of a rapidly selected 4.3kb transposon-containing structural variation with a P450-based resistance to pyrethroids in the African malaria vector Anopheles funestus
Source: PLoS Genet. 2024 Jul 29;20(7):e1011344. doi: 10.1371/journal.pgen.1011344 (PMC11309504; doi:10.1371/journal.pgen.1011344)
Supplement: S3 Table — (DOCX) [file pgen.1011344.s009.docx]

**S3 Table:** **Table S3:** Tajima’s D values across the rp1 locus. The intergenic region between *CYP6P5* and *CYP6P9b* genes is shown in bold at position 8556001 to 8557000.

| **Start** | **End** | **Uganda_Td** | **Cameroon_Td** | **Ghana_Td** |
| --- | --- | --- | --- | --- |
| 8525001 | 8526000 | -2.60 | 0.31 | 2.26 |
| 8526001 | 8527000 | -1.66 | 0.61 | 2.12 |
| 8527001 | 8528000 | -1.44 | -0.16 | 2.00 |
| 8528001 | 8529000 | -2.06 | -0.58 | 1.63 |
| 8529001 | 8530000 | -1.47 | 0.53 | 1.42 |
| 8530001 | 8531000 | -0.46 | -0.82 | 0.03 |
| 8531001 | 8532000 | -1.03 | 0.36 | 1.76 |
| 8532001 | 8533000 | N/A | 0.15 | 1.88 |
| 8533001 | 8534000 | -1.64 | -0.43 | 1.99 |
| 8534001 | 8535000 | -2.32 | -0.02 | 1.25 |
| 8535001 | 8536000 | -0.34 | -0.52 | 1.39 |
| 8536001 | 8537000 | 0.17 | -0.25 | 1.71 |
| 8537001 | 8538000 | 0.10 | -1.26 | 0.10 |
| 8538001 | 8539000 | 0.24 | -1.24 | -0.17 |
| 8539001 | 8540000 | -0.70 | -0.23 | 0.82 |
| 8540001 | 8541000 | -0.35 | -1.08 | 1.89 |
| 8541001 | 8542000 | 1.10 | -1.05 | 1.32 |
| 8542001 | 8543000 | 0.71 | -0.55 | 0.98 |
| 8543001 | 8544000 | -1.66 | 0.78 | 0.15 |
| 8544001 | 8545000 | N/A | 0.47 | 0.92 |
| 8545001 | 8546000 | -1.49 | -0.13 | -1.18 |
| 8546001 | 8547000 | 0.25 | 0.59 | 0.39 |
| 8547001 | 8548000 | -2.01 | 0.58 | 1.66 |
| 8548001 | 8549000 | 0.24 | 0.96 | 1.16 |
| 8549001 | 8550000 | -0.87 | 0.95 | -0.15 |
| 8550001 | 8551000 | -1.31 | -0.65 | 0.53 |
| 8551001 | 8552000 | -0.27 | -0.44 | -0.55 |
| 8552001 | 8553000 | -0.83 | -1.07 | 1.53 |
| 8553001 | 8554000 | -0.80 | 0.16 | 0.32 |
| 8554001 | 8555000 | -1.53 | 0.61 | -0.35 |
| 8555001 | 8556000 | -2.10 | 0.62 | -0.60 |
| **8556001** | **8557000** | **N/A** | **0.06** | **-0.54** |
| 8557001 | 8558000 | -2.17 | 0.08 | -1.33 |
| 8558001 | 8559000 | -0.70 | 0.42 | -1.30 |
| 8559001 | 8560000 | -2.12 | 0.16 | -0.87 |
| 8560001 | 8561000 | 0.94 | 0.04 | -0.55 |
| 8561001 | 8562000 | -0.20 | 0.18 | -0.94 |
| 8562001 | 8563000 | -1.90 | -0.01 | 0.07 |
| 8563001 | 8564000 | 1.26 | -0.41 | 0.14 |
| 8564001 | 8565000 | -2.07 | 1.49 | 0.13 |
| 8565001 | 8566000 | -1.59 | 0.12 | 0.38 |
| 8566001 | 8567000 | N/A | 0.07 | -0.54 |
| 8567001 | 8568000 | -2.28 | 0.82 | 0.56 |
| 8568001 | 8569000 | -1.89 | 0.76 | 0.28 |
| 8569001 | 8570000 | -1.43 | 0.54 | -0.13 |
| 8570001 | 8571000 | -1.88 | 0.43 | 0.59 |
| 8571001 | 8572000 | -1.74 | 0.38 | -0.19 |
| 8572001 | 8573000 | -1.27 | 0.30 | 0.06 |
| 8573001 | 8574000 | -1.41 | 0.43 | 0.46 |
| 8574001 | 8575000 | -1.57 | -0.27 | 0.08 |
