# Supplementary material for: Association of a rapidly selected 4.3kb transposon-containing structural variation with a P450-based resistance to pyrethroids in the African malaria vector Anopheles funestus
Source: PLoS Genet. 2024 Jul 29;20(7):e1011344. doi: 10.1371/journal.pgen.1011344 (PMC11309504; doi:10.1371/journal.pgen.1011344)
Supplement: S4 Table — (DOCX) [file pgen.1011344.s010.docx]

| Transcription Factor Name | Transcription Factor ID | Start Position | End Position | Strand | Core Match Score | Matrix Match Score | Sequence |
| --- | --- | --- | --- | --- | --- | --- | --- |
| Ahr::Arnt | MA0006.1 | 183 | 188 | 1 | 1 | 0.99 | AGCGTG |
| Ahr::Arnt | MA0006.1 | 3449 | 3454 | 1 | 1 | 1 | TGCGTG |
| Ahr::Arnt | MA0006.1 | 4283 | 4288 | -1 | 1 | 1 | TGCGTG |
| Ahr::Arnt | MA0006.1 | 106 | 111 | -1 | 1 | 1 | TGCGTG |
| MAFG | MA0659.2 | 4276 | 4290 | 1 | 1 | 0.884 | ACGTCAGCACGCATT |
| MAFG | MA0659.2 | 936 | 950 | -1 | 1 | 0.866 | TCCTCAGCAAAGCAT |
| MAFG | MA0659.2 | 115 | 129 | -1 | 1 | 0.936 | AGATCAGCAGTTAAA |
| MAFG | MA0659.2 | 4276 | 4290 | 1 | 1 | 0.884 | ACGTCAGCACGCATT |
| MAFG | MA0659.2 | 936 | 950 | -1 | 1 | 0.866 | TCCTCAGCAAAGCAT |
| MAFG | MA0659.2 | 115 | 129 | -1 | 1 | 0.936 | AGATCAGCAGTTAAA |
| MAFF | MA0495.3 | 4276 | 4291 | 1 | 1 | 0.861 | ACGTCAGCACGCATTC |
| MAFF | MA0495.3 | 114 | 129 | -1 | 1 | 0.913 | AGATCAGCAGTTAAAT |
| Mafb | MA0117.2 | 117 | 128 | 1 | 1 | 0.936 | TAACTGCTGATC |
| Mafb | MA0117.2 | 938 | 949 | 1 | 1 | 0.91 | GCTTTGCTGAGG |
| Mafb | MA0117.2 | 1377 | 1388 | 1 | 1 | 0.855 | TCTAAGCTGAAT |
| Mafb | MA0117.2 | 1426 | 1437 | 1 | 1 | 0.896 | AAAAGGCTGATA |
| Mafb | MA0117.2 | 1905 | 1916 | 1 | 1 | 0.857 | CCAGAGCTGATT |
| Mafb | MA0117.2 | 2159 | 2170 | 1 | 1 | 0.867 | TTTGAGCTGATA |
| Mafb | MA0117.2 | 2543 | 2554 | 1 | 1 | 0.865 | TTCCAGCTGAGT |
| Mafb | MA0117.2 | 4277 | 4288 | -1 | 1 | 0.942 | TGCGTGCTGACG |
| Mafb | MA0117.2 | 1587 | 1598 | -1 | 1 | 0.864 | CGATCGCTGAAA |

S4 Table: Putative transcription factors binding sites in 4.3kb structural variants generate by CiiiDER software
