# Supplementary material for: Association of a rapidly selected 4.3kb transposon-containing structural variation with a P450-based resistance to pyrethroids in the African malaria vector Anopheles funestus
Source: PLoS Genet. 2024 Jul 29;20(7):e1011344. doi: 10.1371/journal.pgen.1011344 (PMC11309504; doi:10.1371/journal.pgen.1011344)
Supplement: S5 Table — (DOCX) [file pgen.1011344.s011.docx]

Table S5: Allelic frequencies of the 4.3kb SV among plasmodium infected and non-infected mosquitoes.

| Plasmodium infection status \genotype | SV+ | SV- |
| --- | --- | --- |
| Non infected | 54.7619 | 45.2381 |
| infected | 37.83784 | 62.16216 |
