# Supplementary material for: Association of a rapidly selected 4.3kb transposon-containing structural variation with a P450-based resistance to pyrethroids in the African malaria vector Anopheles funestus
Source: PLoS Genet. 2024 Jul 29;20(7):e1011344. doi: 10.1371/journal.pgen.1011344 (PMC11309504; doi:10.1371/journal.pgen.1011344)
Supplement: Attachment — Submitted filename: Response to reviewers.docx [file pgen.1011344.s013.docx]

**A letter containing a point-by-point response to the reviewer and editor comments and a description of the changes made in the revised version of manuscript**

Date: February 15, 2024

The Academic Editor and Editor-in-Chief,

PLOS Genetics

We are very grateful for the thorough review done on our manuscript (PGENETICS-D-23-00856). We have addressed all the concerns of the reviewer and editors and hope that this improved version of the manuscript meets the expectation of your journal. We have indicated all changes made on the revised manuscript with track changes. The comments were very helpful and we are looking forward to receiving a favorable response.

Yours sincerely,

**Mugenzi Jean Leon**

**(Corresponding author)**

**Reviewer 1**

Comment

In the results it would be useful to have just a little more detail on the transposon implicated in resistance. For example what features) does it contain (in addition to the two ORFs described), are terminal inverted repeats present (if so are these perfect/imperfect)…can the target site duplication site be identified etc.

**Comment** : Line 309: The English in the first section of the results is a little clumsy and should be rephrased to be clearer

Response:

Thank you for your valuable feedback. We have carefully revised the English in the first section of the results to enhance clarity, as suggested. We hope the improvements meet your expectations, and we appreciate your continued guidance throughout the review process.

Reviewer #2:

Retrotransposon identification

**Comment**:
• Authors confuse ORF, retrotransposon and retrovirus concepts throughout the MS Λ. The gag and pol ORFs identified in the 4.3 kb SV belong to one retrotransposon, not several. Unlike retroviruses, many retrotransposons do not all contain an env gene (e.g. see the yeast TY1 retrotransposon). Known retrotransposons, in their genomic DNA version, all have either direct terminal repeats (U3/R/U5, e.g. TY1 again), or a 3’ polyA tail (like human LINE elements). Here, such structures have not been searched for.
• Protein homologies should help to clarify the missing retrotransposon classification. Include multiple alignments to gag gene and reverse transcriptase domain of retroelements known to move, from the LTR and polyA classes. This could also be performed for the FUMOZ copy -or copies.
• The described SV may be a full, autonomous retrotransposon or a truncated one, but at any rate, because a target site duplication is shown to exist, the SV inserted through a transposition mechanism, and authors can safely call it a retrotransposon, which would make things clearer. I encourage them to change terminology throughout the MS.

**Response:**

Methods:

We uploaded the high-coverage region of the insertion sequence to RepBase (Name: TE-1_AFu) and annotated it using the CENSOR and InterProScan webservers (Kohany et al; 2006, Jones et al 2014).

Results:

We have added a description of the structures of the retrotransposon.

The insertion sequence encodes a retrotransposon in the Jockey superfamily of long interspersed nuclear elements (LINEs). Jockey retrotransposons do not contain long terminal repeats (nonLTR) and are restricted to arthropods in their distribution (Metcalfe and Casane 2014). The shorter of the two ORFs at 394 amino acids (aas) encodes a zinc finger domain, while the longer domain (906 aas) encodes endonuclease/exonuclease/phosphatase and reverse transcriptase domains in line with other Jockey elements (Metcalfe and Casane 2014). A CENSOR search if the RepBase database revealed that a Jockey retrotransposon in *An. gambiae* (Ag-Jen-1) as the only similar sequence with a blast alignment score (“bit”) of 3,997 and similarity of 0.67. There is a run of 15 Adenine bases in the 3’ UTR of the insertion sequence consistent with a polyA tail.

**Comment:**

• Submit transposon sequence to a database like repBase

**Response:**

The transposon sequence has been submitted to repBase.

**Comment :**

**qRT-PCR**
• The two CYP6P9a/b genes are highly homologous and several kb apart, casting some doubt on the conclusion that both genes are up-regulated in SV+/SV+ individuals. Specificity controls, for instance with cloned a and b genes should be shown or mentioned. Showing melt curves with different Tm is another option.

**Response:**

We concur with the reviewer's observation that the CYP6P9a/b genes exhibit significant similarity. Consequently, the reverse primers were strategically designed to target the 3´UTR of each gene where there are less similarities, enhancing specificity. The sequences of the primers have been added to the supplementary file **Table S2**

Comment :

I could not even find a list of used primers, nor PCR conditions…

Response:

The primers and conditions have been added. The primer sequences can be found in Table S2 of the supplementary file.

Comment :

Experimental conditions and control data should be included to convince the reader of the observations, and if conclusions are maintained, they should be fully discussed. To my eyes, the negative conclusion on CYP6P5 needs to be tuned down because all expression levels are very low.

Response:

We agree with the reviewer´s comment there observed expression levels are not superhigh. We have indicated this is the Manuscript Line- 549-552

“Quantitative real-time PCR performed on pools of each of the 3 genotypes (SV+/SV+, SV+/SV- and SV-/SV-) relative to FANG revealed moderate expression of nearby genes with increased expression of *CYP6P9a* (downstream) and *CYP6P9b* (immediately downstream) but not of *CYP6P5* (upstream) in the SV+/SV+ pool only (Figure 4A)”.

We have also alluded to the potential impact of this insertion on other genes located at a distance.

Line 667 “ The RNA sequencing data also unveiled additional P450s exhibiting higher expression levels compared to the ones under our investigation. Therefore, there is a potential that this insertion may be contributing to the increased expression of other genes located at a distance.”

Comment :

An intriguing outcome of this experiment is that the P450 expression phenotype is fully recessive (overexpression in SV+/SV+ only) while the resistance phenotype is consistently semi-dominant (Fig. 3). This difficulty should be discussed.

Response:

Thanks for pointing this out we have added this to the discussion.

Line 667-680 “The findings indicate a fully recessive P450 expression phenotype, characterized by overexpression exclusively in SV+/SV+ individuals. In contrast, the resistance phenotype consistently exhibits semi-dominance. This semi-dominance in the resistance phenotype may be attributed to a reduced exposure time of 30 minutes. Further investigations, involving an extended exposure time of 90 minutes, might unveil that the resistance phenotype is actually recessive, necessitating the presence of two alleles.”

Comment:

• The ‘2-ΔΔCT method incorporating PCR efficiency’ (line 261) is self-contradictory. Taking into account PCR efficiencies is a E-ΔΔCT method, where E is the PCR efficiency deduced from a dilution series : E<2 .
• How double normalization is achieved must be explicited¬¬

Response:

Thank you for your insightful comment. We appreciate the clarification you provided regarding the '2-ΔΔCT method incorporating PCR efficiency.' You are correct in pointing out that considering PCR efficiencies transforms it into an E-ΔΔCT method, where E represents the PCR efficiency derived from a dilution series, and it's essential that E is less than 2.

We did a correction for PCR efficiency [CtE = Ct*((log(E+1)/(log(2))] before performing the 2-ΔΔCT analysis which is known to improve the accuracy and reliability of results, especially when dealing with genes that may have different amplification efficiencies.

Comment:

**Putative binding sites for transcription factors (TFs)**
• Not only the 4.3 kb SV but the whole intergenic regions (CYP6P5-CYPP9b and CYPP9b-CYP6P9a) should be included in such a hunt.

Response:

Our objective in this study was to examine the presence of potential binding sites within the inserted segments. This aimed to identify additional cis-regulatory elements contributed by this insertion to the surrounding region.

Comment:

• Identified candidate target sites should be drawn on a map (possibly Fig1C) and homologies presented in a supplementary Figure.

Response

Comment:

• The claimed up-regulation of two genes separated by several kb would imply that some TF act in an enhancer, i.e. far away from the TATA box, rather than in a promoter. This is not discussed. Is there evidence for this in the literature for Ahr/ARNT or MAF TFs?

Response:

We did find any study that looked at this.

**Comment:**

Interference between SV and Plasmodium infection

• A remarkable result, to be mentioned in Abstract.

**Response:**

We have incorporated the suggested addition into the abstract.

Line 42-44 “ An inverse correlation was observed between the 4.3kb SV and malaria parasite infection, indicating that mosquitoes lacking the 4.3kb SV were more frequently infected compared to those possessing it.”

**Comment:**
• Association is weak, although significant, which requires showing odd ratios in a Table

**Response:**

A table showing the odd ratios for the different genotypes has been added

Table 3.

**Table 3:** Associations between 4.3kb SV genotypes and likelihood of not being infected by Plasmodium parasite.

| **Comparison** | **OR^a^** | **P value** | **CI^b^** |
| --- | --- | --- | --- |
| SV+/SV+ vs SV-/SV- | 4.1 | 0.0017 | 1.7-9.9 |
| SV+/SV- vs SV-/SV- | 4.2 | 0.0001 | 2.1-8.68 |
| SV+/SV+ vs SV+/SV- | 1 | 0.99 | 0.47-2.1 |
| SV+ vs SV- | 2 | 0.02 | 1.13-3.51 |

**Comment:**

Fig 1A:
• Picture is fuzzy, genomic scale cannot be read etc… Change for a high definition pic imperatively.

• Not everybody is a IVG user… Precise the meaning of colored reads in the caption
• Precise what should be seen in circles.
• Uganda split reads at the retrotransposon insertion site could be highlighted, or at least mentioned.

**Response:**

We have made the requested adjustments.

**Comment:**
Fig1C:
• switch also to a high definition picture.
• Precise coordinates of the intergenic region on the reference genome.
• Remove ‘retrotransposonS’, add gag and pol, plus polyA or LTRs if any.
• The position and orientation of the CYP6P9a gene should be drawn, or at least mentioned in Legend.
• Use the same orientation for the top and bottom panels.
• Precise if bottom panel is the reconstituted 5.4 kb, SV containing intergenic region. It does not look like the reference genome, but is just below Fig 1A...
• Place symbols to indicate the relevant TF binding sites identified
**Response:**

We have adjusted the figure as requested, but indicating the relevant TFs was not easy as there were many we have added a table indicating their locations in the supplementary file.

**Comment:**
Fig 2
• For Fig 2A, precise the pedigree of assayed animals: captured individuals, or their offspring ? It is obscure why the three genotypes are not or cannot be displayed like in Fig 2C, E, F. Avoid “population” in Legend, be more precise

**Response:**

We have added the pedigree to the legend and replaced figure of alleles with that of phenotypes.

**Comment:**
• Give sample numbers.

**Response:**

The number of samples have been added to figure 2A.

**Comment:**
• Fig2B and D: I could not see usefulness, next to Fig C and E that are perfectly illustrative. Note Fig 2F has no such an appendice, which is fine. Does SV+ refers to the SV allelic frequency, or to the proportion of individuals with at least one copy ?
• Write Cameroun ahead of Tibati, for consistency.

**Response:**

We have removed figure 2C and E and moved them to the suplimentary file

**Comment:**
Fig 3
• Fig 3 A,C,E, G,I : stack Alive above Dead consistently. Precise the total number of mosquitoes in each genotype

**Response:**

We have rectified this and added the number of mosquitoes genotyped.

**Comment:**
• Fig 3A and 3C Legend, give insect pedigree: F1 Gounougou 2018 females pooled from several mothers?

**Response:**

This has been added to the legend.

**Comment:**
• it would be good to adopt the same graphic code as Fig 4B, i.e. 2 collumns with 3 genotypes stacked.

**Response:**

Based on the 3 by 2 comparison done in the manuscripts keeping the graphs in this form makes it easy for the reader .

**Comment:**
• Remove Fig 3 B,D, F ,H, or explain their added value,

**Response:**

These figures has been moved to the supplementary file

**Comment:**
Figure 4
• In Fig 4A, the relative expression should be readable as a ratio between genotype of interest and FANG parent. The fold change to FANG must be given for one of the 3 genes and one of the 3 genotypes.

**Response:**

The fold change of Fang has been added to the figure 4A

**Comment:**
• Remove Fig 4 C or explain its added value: Fig 4B seems sufficient

**Response:**

This has been removed.

**Comment:**

Table 1
• indicate net active molecule content.

Response: this has been indicated Line 169-174

**Comment:**

• Include the four net types mentioned in Results

**Response:**

We included only those with pertinent results. As Permanent 3.0 all mosquitoes exposed were dead.

**Comment:**
• indicate mosquito numbers and overall mortality %,

**Response:**

This was indicated in the Text.

**Comment:**
• Does SV+ vs SV- refers to a ratio of alleles, or to the proportion of individuals with at least one copy?

**Response:**

This represent the proportion of individuals with one copy.

**Comment:**
• Explicit H0 (presumably no phenotype-genotype association).
**Response:**

We have indicated this in the data analysis section

Minor points

Abstract

Comment:
• Abstract should include the identification of TFs within SV, as well as SV role in reducing Plasmodium infection rate.

**Response:**

We have incorporated the identification of transcription factors within this structural variant (SV) and the SV's impact on diminishing the Plasmodium infection rate into the abstract.

**Line 40-42:** "Within this structural variant (SV), we identified putative binding sites for transcription factors associated with the regulation of detoxification genes."

**Line 42-44:** “An inverse correlation was observed between the 4.3kb SV and malaria parasite infection, indicating that mosquitoes lacking the 4.3kb SV were more frequently infected compared to those possessing it.”

**Comment:**
• L37: Abstract should avoid stat test

**Response:**

The stat test has been removed.

**Comment:**
• Line 39, expression, not overexpression; tune down the negative conclusion with CYP6P5.

**Response:**

This has been corrected.

**Comment:**

• L39-41, move sentence earlier in the flow of ideas

**Response:**

Thanks for pointing this out the sentence has been moved. **Line X**

A marked and rapid selection was observed with the 4.3kb-SV frequency increasing from 3% in 2014 to 98 % in 2021 in Cameroon,

**Comment:**

• The Introduction ends with another version of the Abstract, rather than stating the aims of the study. The Wondj et al 2009 paper does not appear in References.

**Response:**

- The conclusion of the introduction has been revised to integrate the study's objective

Line 97-100 In the current study, we investigated whether a structural variant insertion in a cytochrome P450 locus can modify the pyrethroid resistance phenotype in *An. funetus*. We discovered a 4.3 kb insertion in the p450s loci rp1, previously identified by QTL mapping, housing two insecticide resistance genes, CYP6P9a and CYP6P9b (Wondji et al., 2009). This insertion was found across Central, East, and West Africa but was absent in southern regions. Monitoring in *An. funestus* populations of Cameroon revealed strong selection on this structural variant, suggesting it may be adaptive or linked to nearby adaptive mutations. Genetic crosses indicated a robust association between this variant and resistance to pyrethroids, as well as overexpression of nearby genes.

- And the reference has been added,

**Comment:**

Introduction
It seems relevant to mention the absence of kdr mutations driving pyrethroid resistance in An. funestus, stressing the importance of metabolic resistance

**Response:**

Thank you for your insightful comment. We have now incorporated the absence of kdr mutations in An. funestus into the introduction, highlighting the significance of metabolic resistance as a key factor driving pyrethroid resistance in this species.

**Line 65-68**: In the context of pyrethroid resistance, mutations primarily associated with its target, the voltage-gated sodium channel, are common. However, in *An. funestus*, no Kdr mutations have been identified . Therefore, pyrethroid resistance in this species is predominantly metabolic in nature.

**Comment:**
Mosquito sample rearing:
• L112-114, several publication dates are earlier than the mentioned captures. Explain.

**Response:**

Thank you for bringing this to our attention. We appreciate your diligence in reviewing our work. The publication dates mentioned in L112-114 refer to studies conducted in those locations as we used samples previously collected in this studies, and we acknowledge the potential for confusion. To address this concern, we have rephrased this sentence in two parts. Firstly talking about the sample that we collected and then those from previous studies.

Line 111-115

where *An. funestus* s.s. is the predominant vector. The selected villages and corresponding years of collection are as follows: Gounougou (9°03′00″N, 13°43′59″E) in 2017, 2020, and 2021; Elende (3°41'57.27''N, 11°33'28.46''E) in 2020; Mibellon (6°46′N, 11°70′E) in 2018 and 2020; Tibati in 2021 (6°28' N, 12°37' E) and Elon 4°13'49"N, 11°36'04"E in 2021.

**Comment:**
• L118. The offspring of the field caught females is called F1. Indicate the approx. number of females captured, and state whether you pooled the offspring of several females to produce F1 or F3s.

**Response:**

- We have mentioned the approximate number of mosquitoes collected per day and gave the numbers for the highest and lowest catch. Line 121-124

“The number of blood-fed *Anopheles* females collected varied from one location to another with an average of 100 per day in Gounougou and Elende and 50 or less in some locations like Tibati.”

- We have also so stated that the F1 were pooled to have the next generation.

**Comment:**

I understand that Fig 2A used directly captured individuals rather than their offspring,if so please precise it in M&M.

**Response:**

Indeed, we utilized F0 samples captured in the field, and this information has been explicitly mentioned in both the text and the legend of Figure 2A.

Line 387-390: The assay was initially tested on Uganda (Tororo, 2014) and Cameroon (Mibellon, 2021) field caught (F_0_) samples containing the insertion and showed a band at 780bp. Further genotyping of Uganda (Tororo 2014 and Mayuge, 2017) field caught (F_0_) mosquito samples confirmed its presence and at a high frequency approaching fixation of the 4.3kb SV in Uganda mosquitoes with 100% frequency in Tororo and 97.83% in Mayuge with no mosquito found homozygous for the wild SV- allele (Figure 2A).

**Comment:**
• L119, precise year.

**Response:**

The year has been added.

Line 127-128: F_1_ mosquitoes from Gounougou (collected in 2018) were used for susceptibility testing and evaluating bed net efficacy.

**Comment:**
• L133-142, pedigree is not clear. Do you refer to field caught mosquitoes or to their F1 offspring as parents for the crosses to FANG ? No capture of males nor virgin females from the field is mentioned. If parents for crosses are named F1 individuals, precise the number of insectarium selfings to produce F3s.

**Response:**

This has been clarified in the text

Line 146-156:

Female F_1_s of field-collected mosquitoes were crossed with male FANG to generate a field x FANG line using about 500 individuals from each. A reciprocal crossing using about 500 female FANG and 750 F_1_ males from the field caught mosquitoes was also generated. Only the female FANG crossed with F_1_ males for the field was successfully established. A restricted cohort of approximately 100 F_1_ individuals was derived from the initial cross, subsequently subjected to intercrossing to generate the F_2_ generation. Approximately 200 F_2_ individuals were obtained and further subjected to crossing and bloodfeeding to produce the F_3_ generation, resulting in a population increase to approximately 600 individuals. This F3 population was utilized for the bioassays. The complete study involved the generation of three distinct lines.

**Comment:**
• L149 and 160, are there any knock down data in the paper ?
**Response:**

We have added the knockdown data to the manuscript.

Line 447: “ The mortality rate for deltamethrin was 49.8±11.6% with a knockdown rate of 94.5%.”

Line 480-481: “ The knockdown rates were 39±6.4% for Olyset, 30.1±7.5% for PermaNet 2.0, 72.9±24.4% for Olyset plus and 100% for PermaNet 3.0.”

**Comment:**

Bioassays
• L155, precise the content of the various insecticide containing nets.

**Response:**

The insecticide content of the nets have been indicated.

Line 168-174: “ Cohorts of 50 mosquitoes were exposed to 25cm x 25cm pieces of the netting of PermaNet 3.0 (Combination of 2.8 g/kg of deltamethrin on side panels and deltamethrin (4.0 g/kg) and PBO (25 g/kg) on roof, manufactured by Vestergaard Frandsen), PermaNet 2.0 (5.5 × 10−5 kg/m^2^ of deltamethrin, manufactured by Vestergaard Frandsen), Olyset (8.6 × 10−4 kg/m^2^ of permethrin, manufactured by Sumitomo Chemical), and Olyset Plus (8.6 × 10−4 kg/m2 of permethrin and 4.3 × 10−4 kg/m2 of PBO, manufactured by Sumitomo Chemical”,

6. SV identification:

**Comment:**

• L165-167, redundant with intro, please delete.

**Response:**

Sentence has been deleted.

**Comment:**
• L168-169. Precise sequencing method and the countries/years of origin

**Response:**

This has been added to the text

Line 187-190: The locations and year of collection are Benin (Kpome, 2015); Ghana (Obuasi, 2014); Cameroon (Mibellon, 2015); Uganda (Tororo, 2014); Democratic Republic of Congo [Kinshasa (2015), Mikalayi (2015]; Malawi (Chikwawa, 2014); Mozambique [Palmeira ( 2016), Morrumbene (2002) and Zambia (Kaoma, 2013) plus the FUMOZ and FANG laboratorz colonies. These samples underwent sequencing using an Illumina HiSeq 2500 platform (2 × 150 bp, paired-end).

**Comment:**
Nucleotide diversity
• L189-193, move to legend of Fig S1

**Response:**

This sentence has been moved to legend of Fig S1.

Line 211- *CYP6P5* and *CYP6P9b* were created using the karyoploteR package (v1.16.0) in R version 4.0.3 (Gel & Serra, 2017)

**Comment:**

Stat tests on genotype segregation
• L298-305. Authors have chosen to explore deviations to expected segregations both by Chi-square tests and by odd ratio stat tests. Please indicate the added value of this double checking. Explicit H0 (presumably no phenotype-genotype association).

**Response:**

- The utilization of both Chi-square tests and odds ratio statistical tests in exploring deviations to expected segregations serves the purpose of robustness and comprehensive analysis. While the Chi-square test provides a traditional approach for examining deviations, the odds ratio statistical test offers an additional perspective, allowing us to assess the strength and direction of association. By employing both methods, we aim to ensure the reliability and validity of our findings, enhancing the overall rigor of the analysis and providing a more comprehensive understanding of the observed deviations in the context of our study.
- The null hypothesis has been explicitly stated

**Line 324:** The null hypothesis (H0) was that there is no statistically significant association between the observed phenotype and the genotype under investigation. Any observed differences in the phenotype are due to random chance or factors unrelated to the genotype being studied.

**Comment:**
Results
• L309-310, precise increase vs what.

**Response:**

We indicated that the increase was noticed in Uganda and not in the other countries.

Line 332: Examining the PoolSeq IGV alignments uncovered a significant spike in coverage within the intergenic region in Uganda in 2014 compared to other locations

**Comment:**
• L310-311, replace ‘transposon insertion position’ by ‘ 9 bp transposon insertion target site duplication’,

**Response:**

This has been replaced Line 332 “Examining the PoolSeq IGV alignments uncovered a significant spike in coverage within the intergenic region in Uganda in 2014 compared to other populations (Figure 1A) corresponding to a 9 bp transposon insertion target duplicationat positions 8,556,411-8,556,420 on Chromosome 2, marked by the sequence “CAAATGTACA.”

**Comment:**

• L344, replace ‘5.4 kb for the open reading frames’ by ‘5.4 kb intergenic region’.

**Response:**

This has been replaced

Line 365 : “ Analysis of the entire 5.4 kb intergenic region revealed the presence of 2 open reading frames corresponding to putative transposable elements.

**Comment:**
• L346-348, ORFs, not transposons. Indicate % of homology

**Response:**

This has been corrected and the percentage identity has been added.

Line 367

BLASTp identified these open reading frames to match a gag-like protein from *Culex pipens* (AAB86424.1) with percentage identity of 29% and a reverse transcriptase-like protein from *Aedes aegypti* (AAA29354.1) with percentage identity of 39%,

**Comment:**
• Line 420-424 describes Fig 3A, a 3 genotypes x2 phenotypes matrix, using a 2x 3 matrix. This cannot be easily followed. Either rewrite, or redraw Fig alike Fig 4B.

**Response:**

We have rephrased the text to match Fig 3A.

Line 450-455: Within the homozygous (SV+/SV+) genotype, 74.4% (32/44) of individuals were found to be alive, while 25.2% (11/43) were deceased (Figure 3A; Table 1). Among those with the heterozygous genotype (SV+/SV-), 22.2% (2/9) were alive, contrasting with 77.8% (7/9) that were not alive. Notably, the majority of individuals lacking the structural variant (SV-/SV-) exhibited a mortality rate of 96% (24/25), with only 4% (1/25) surviving (Figure 3A; Table 1).

**Comment:**
• Line 424_436: the Result section just parrots data available in Table1, making reading quite reader-unfriendly. The basic conclusion -here, that survival probability increases with SV copy number- is not drawn. This unfortunate style, with conclusions postponed to Discussion, is used repeatedly for all genotype/phenotype association experiments, and is a real pain in the neck: please shorten this section and refers to Table 1.

**Response:**

We have addressed this concern by streamlining the presentation of data in lines 450-468, emphasizing the main conclusion regarding the positive correlation between survival probability and SV copy number. We have also make explicit references to Table 1 for detailed information.

**Comment:**

- Separating Figures for sections 5.1 and 5.2 would also help.

**Response:**

We have separated the figures and send some to the supplementary files.

**Comment:**
• Line 444 or anywhere earlier, precise LLIN active ingredient compositions, including possible top/side differences.

**Response:**

The insecticide content of the nets have been indicated.

Line 168-174: “ Cohorts of 50 mosquitoes were exposed to 25cm x 25cm pieces of the netting of PermaNet 3.0 (Combination of 2.8 g/kg of deltamethrin on side panels and deltamethrin (4.0 g/kg) and PBO (25 g/kg) on roof, manufactured by Vestergaard Frandsen), PermaNet 2.0 (5.5 × 10−5 kg/m^2^ of deltamethrin, manufactured by Vestergaard Frandsen), Olyset (8.6 × 10−4 kg/m^2^ of permethrin, manufactured by Sumitomo Chemical), and Olyset Plus (8.6 × 10−4 kg/m2 of permethrin and 4.3 × 10−4 kg/m2 of PBO, manufactured by Sumitomo Chemical”,

**Comment:**
• Line 452, avoid “samples’ when ‘individuals’ can be used ( a comment also valid elsewhere).

**Response:**

Thank you for your valuable feedback. We appreciate your suggestion to replace "samples" with "individuals" in line 452, and we have incorporated this improvement. Additionally, we have carefully review the manuscript to identify and address similar instances where using "individuals" is more appropriate than "samples.

**Comment:**
• Line 460-461, if SV+ homo- ands heterozygotes both survive 100%, how can Odd Ratio be ≠ 1 ??

**Response:**

There was a mistake in the calculations this has been corrected. The odd ratio =1

Line 487-489:

“In comparison, SV+/SV+ homozygote mosquitoes and heterozygote (SV+/SV-) mosquitoes exhibited a significantly higher likelihood of survival following PermaNet 2.0 exposure when contrasted with mosquitoes entirely lacking the 4.3kb SV (SV-/SV-) (OR: 201; CI: 12.15-3325.23; P = 0.0002).”

Table 1.

**Comment:**
• Line 472-474, no significant ¬≠ despite p=0.04 ?

**Response:**

This has been corrected.

Line X: There was a marginally significant differences in surviving exposure to Olyset Plus between the SV+/SV+ and the SV-/SV- genotype (OR: 1.8; CI: 1.0 to 3.3; P = 0.04).

**Comment:**
• Line 477-478, borderline significance, p=0.05

**Response:**

We have stated that the results were marginally significant.

**Line X** A weak significant survival likelihood was observed when comparing SV+ and SV- (OR: 1.8; CI: 1.03 to 3.32; P = 0.04).

**Comment:**
• Line 484, remove Type II

**Response:**

This has been removed line X: Results from deltamethrin (0.05%) bioassay at F_3_ exposed

**Comment:**
• Line 485, remove “showing that the crossing was still resistant”, or show FANG control

**Response:**

This has been removed line X Results from deltamethrin (0.05%) bioassay at F_3_ exposed

**Comment:**
• Line 487, use past tense
**Response:**

This sentence has been changed to the past tense. Line X SV+ was significantly associated with survival to deltamethrin exposure (OR: 19.4; CI: 9.5 to 39.6; P< 0.0001),

**Comment:**
qRT-PCR,
• Line 516 or earlier, the genomic architecture of the CYP6P5, CYPP9b, and CYP6P9a segment, including orientation and distances, should have been described or illustrated – possibly in Fig 1C.

**Comment:**
• Line 526, remove ‘could’ and ‘further’, both are misleading

**Response:**

These words have been removed.

Line 557-558: This indicates that possessing 2 copies of this 4.3kb SV enhances the expression of these genes.

**Comment:**
• Line 530, four 4 announced binding sites and only 3 mapped.
• Line 531-2, 14 sites and none mapped

**Response:**

To correct this, we have put the locations of the binding sites in table S3.

**Comment:**
Interference between SV and Plasmodium infection
• L542-548, move Odd ratios to a table, and summarize conclusions in Results.

**Response:**

We have summarized the Odds ratios in a table as suggested, Table 3.

**Comment:**

Discussion
• Line 566-572 learn about retrotransposons and rephrase: gag and pol only- no env- for most LTR retrotransposons

**Response:**

We have added more details about the LTR as describe above.

**Comment:**
• Line 571 & 574, Nevedova et al papers absent from reference list. The presence of putative TF binding sites within the transposon should be recalled

**Response:**

We have rectified this and discussed the presence of putative TF binding sites. **Line X**

**Comment:**

• Line 572-575, the relevance of gagr is obscure

Response:

We tried to describe the structures present in the transposon.

**Comment:**
• Line 576-578. The probability that a retrotransposon inserts twice at the very same place seems small enough for this option not to be formulated.

**Response:**

The reviewer's observation is accurate; we have revised the sentence in line 607-609.

Therefore, we hypothesized that this SV spread from Uganda to Cameroon as the likelihood of a retrotransposon inserting twice at precisely the same location appears to be relatively low.

**Comment:**

This discussion about Cameroon is repeated for Ghana and Benin much later, lines 600-604: why separate ?

**Response:**

We have moved these sentences upwards with the discussion about Cameroon.

**Line 609-611:** Genotyping of recently collected samples for the 4.3kb SV revealed its presence in West Africa in Ghana and Benin at low frequencies, suggesting that this resistance allele is migrating westward.

**Comment:**
• Line 591: syntax?

**Response:**

This has been corrected line 622 The high frequencies observed exhibit a pattern akin to the 6.5 kb structural variant previously identified in *An. funestus* populations from southern Africa, specifically in Malawi and Mozambique (Mugenzi et al., 2020). This variant was correlated with an observed increase in resistance to deltamethrin and permethrin in field populations.

**Comment:**
• Line 594: upstream of what ?

**Response:**

We have indicated that is transposon is found upstream of a cytochrome P450 *CYP6aa1*

Line 627-630: Similarly, in *An. gambiae*, a partial Zanzibar-like transposable element (TE) was identified upstream of *CYP6aa1* in association with two other mutations (nonsynonymous point mutation in *CYP6P4* (I236M) and a duplication of *the CYP6AA1* gene) in Uganda populations at high frequency and shown to have spread to Kenya, the Democratic Republic of Congo and Tanzania (Njoroge et al., 2022).

**Comment:**
• Line 607: ORFs, not transposons Λ

**Response:**

This has been corrected.

**Comment:**
• L607-611: syntax ?

**Response:**

This has been corrected.

**Comment:**
• Line 611-617: sentences belonging to Results

**Response:**

This sentence has been deleted.

**Comment:**
• Line 617-619: the claimed difference between Type I and II pyrethroids seems to contradict overlapping CI in Table I.

**Response:**

This statement has been deleted.

**Comment:**
• Interference between SV and Plasmodium infection
• I hope the trivial following explanation can be discarded: could it be that the higher frequency of SV in the sampling zone would match a patchy pattern of high vs low Plasmodium infection rates, generating a human high vs low use of pyrethroids, and selecting in turn an overlapping pattern of SV frequencies ?

**Response:**

Thank you for your input; however, this explanation cannot account for the observed pattern, given that the genotyped mosquitoes were from the same region with high Plasmodium infection rates, and the bednet usage in the area was consistent.

Reviewer #3:

**Comments:**

Line 142. This line was maintained until the F3 generation when bioassays were performed. – How was it maintained? No additional crosses? Please clarify.

Response:

We have added more details

Line 150-153: A restricted cohort of approximately 100 F_1_ individuals was derived from the initial cross, subsequently subjected to intercrossing to generate the F_2_ generation. Approximately 200 F_2_ individuals were obtained and further subjected to crossing and bloodfeeding to produce the F_3_ generation, resulting in a population increase to approximately 600 individuals.

**Comments:**

Line 146 exposed to the insecticide(s) for either 30 minutes or 1 hour – why 2 time points?

Response:

line 159: The WHO protocol recommends 1 hour exposure but when mosquitoes are crossed with susceptible laboratory strain there is a reduction in resistance as most of the time resistance is recessive. Hence 30 minutes was for the crossed mosquitoes. We have indicated this in the manuscript

**Comments:**

Line 228 – specify final concentration of primers not volume

Response:

The concentration of the primers has been added

Line 247: 0.75µl of 25mM MgCl_2_, 0.12µl of 25mM dNTPs, 0.5µM of each primer, 0.12µl of Kapa

**Comments:**

Line 236 Was F1 or F3 used in the assay? From previous description it seems F3 were used. Please clarify.

Response:

We employed homozygous field F1s carrying this structural variation and laboratory colonies without the structural variation as our positive and negative controls, respectively. This was done to assess the reliability of our assay.

**Comments:**

297 Data analysis – More details are needed on how odds ratios were calculated and generally on the statistical analyses conducted. More details will allow better reproducibility across other researchers.

**Response:**

We have added more details on how the odd ratios were performed.

Line 319: The proportion of each genotype in the alive and dead was determined then used for the odds ratio calculations. The proportion of the alive individuals were considered as subjects with positive outcome while the dead were considered as subject with negative outcome.

**Comments:**

Figure 1 A is not completely clear, consider making it more accessible

**Response:**

We have improved the quality of figure 1.

**Comments:**

Line 336 To sequence the full fragment, four additional sequencing primers were used

337 (Table S1). – include protocol in methods or supplementary material,

**Response:**

This primers were sent with the plasmid to GENEWIZ for sequencing the full insert we did not do any extra PCR with this primers.

**Comments:**

Figure 1 B is not described in the text, this should be included

**Response:**

This has been corrected

**Comments:**

Line 368 Genotyping of the *An. funestus* FUMOZ and FANG lab colonies showed that this

structural variant was absent from those colonies – Can these results be added in Figure 2A?

**Response:** the lab colonies were not included as we wanted to compare the field populations.

**Comments:**

Statistical analysis describing tests showed in figure 3B, D, F, H should be more clearly detailed.

**Response:**

We calculated the proportions of each allele and used them to calculate the odd ratio later.

**Comments:**

Line 494 males (fixed for the SV+) crossed with FANG females (without this SV) at F3 – This (Mibellon) was not described in the methods (only Elende was described); please add to the methods,

**Response:**

Thanks for pointing this out, this has been added to the methodology.

Line 319-320:

The F_1_ of field-caught mosquitoes from Elende and Mibellon were crossed with the FANG *An. funestus* colony to allow segregation of the resistance allele already at a very high frequency in the population

**Comments:**

Details of statistical analysis used to test gene expression was not included and should be described

**Response:**

We have added a statement about the statistical test used to compare the qPCR data.

Line 327-328: Student's t-test was used to compare the means of data obtained for qPCR to determine the level of significance.
