# Supplementary material for: Association of a rapidly selected 4.3kb transposon-containing structural variation with a P450-based resistance to pyrethroids in the African malaria vector Anopheles funestus
Source: PLoS Genet. 2024 Jul 29;20(7):e1011344. doi: 10.1371/journal.pgen.1011344 (PMC11309504; doi:10.1371/journal.pgen.1011344)
Supplement: Attachment — Submitted filename: Response to reviewer.docx [file pgen.1011344.s014.docx]

**A letter containing a point-by-point response to the reviewer and editor comments and a description of the changes made in the revised version of manuscript**

Date: June 4, 2024

The section editors,

PLOS Genetics

Dear Prof. Giorgio Sirugo and Prof Justin Fay,

We are very grateful for the thorough review done on our manuscript (PLOS Genetics (PGENETICS-D-23-00856R1). We have addressed all the concerns of the reviewer and hope that this improved version of the manuscript meets the expectation of your journal. We have indicated all changes made on the revised manuscript with track changes. The comments were very helpful and we are looking forward to receiving a favorable response.

Yours sincerely,

**Mugenzi Jean Leon**

**(Corresponding author)**

**Responses to reviewers’ and editors’ comments**

**Reviewer 2**

Inflated claims

**Comment :**

In the absence of direct evidence, the claim in the title should therefore be tuned down: association, not driving.

**Response**

title has been changed from “A rapidly selected 4.3kb transposon-containing structural variation is driving a P450-based resistance to pyrethroids in the African malaria vector *Anopheles funestus”* to “Association of a rapidly selected 4.3kb transposon-containing structural variation with a P450-based resistance to pyrethroids in the African malaria vector *Anopheles funestus.”*

**Comment** :

Idem in the abstract, line 26-27, ‘we demonstrate that the transposon drives resistance…’,

- We have replaced drives with associated **L26-28**

Here, we demonstrate that a 4.3kb transposon-containing structural variation is associated with pyrethroid resistance in central/eastern African populations of the malaria vector *Anopheles funestus*.

**Comment 3:**

*and in the conclusion, L723-4.*

**Response:**

This has been modified. L652-653

- This study shows how genetic variation, such as transposable elements are linked to adaptive changes and rapidly selected in the mosquitoes carrying them.

**Comments**

Another unproven claim is the link between CYP6P9 overexpression and resistance in the studied populations. There are two unsuspected findings:

**Comment 4:**

1) The recessive overexpression of the CYP6P9 genes in the presence of the 4.3 transposon insertion, independently of the resistance phenotype (Fig 4A). This is reminiscent of the transvection phenomenon in Drosophila, which is worth mentioning. For this point, future reporter gene studies in transgenic mosquitoes should be very informative.

2) The semi-dominant resistance phenotype (Fig 3). A semi-dominant or recessive resistance phenotype (L693-6) is not an absolute property but can vary with the abiotic conditions. Constitutive gene expression data, on the other hand, should only vary with the genotype. The question is not to find conditions in which a semi-dominant phenotype will be hidden, but to explain it when it is found, and results fit only partially, at best, with the ‘more CYP6P9 expression makes more resistance’ explanation (Discussion headline, lines 667-668h). Dissecting the causal chain would require bioassays on transgenic mosquitoes overexpressing CYP6P9a or b in a SV- context, rather than SV-luciferase gene fusions.

To my view, this section of the Discussion (lines 667-696) deserves rewriting.

**Response:**

This section of the manuscript has been revised as suggested by the reviewer.

L590-625

**Comment 5:**

Data mentioned but not shown.

The 4.3 kb SV sequence should be deposited in a public database. It is said to be done, but I could not find it. Please indicate on which line.

**Response:**

Thanks for highlighting this, we have added the accession number in the Data accessibility section**.**

“ The sequences of the 5.3kb intergenic region of *CYP6P5* and *CYP6P9b* have been submitted to GenBank accession number: OR000399. The GenBank accession number of the 4.3kb SV is BK068115.”

**Comment:**

L321, the sequence of the FAM and HEX probes should be indicated.

**Response:**

The sequences of the probes have been added.

**Line 318-323: “**The primers Plas-F (5´-GCT TAG TTA CGA TTA ATA GGA GTA GCT TG-3´) and Plas R (5´-GAA AAT CTA AGA ATT TCA CCT CTG ACA-3´) were used together with two probes tagged with fluorophores: Falcip+(TCT GAA TAC GAA TGT C) labelled with FAM to detect *Plasmodium falciparum*, and OVM+ (CTG AAT ACA AAT GCC) labelled with HEX to detect *Plasmodium ovale*, *Plasmodium vivax,* and *P. malariae*.”

**Comment:**

L406 SV frequency data for Mayuge not shown

**Response:**

SV frequency data for Mayuge has been added to figure 2a

**Comment:**

**L414-5 SV frequency data for FANG and FUMOZ lines are not shown**

**Response:**

SV frequency data for FUMOZ and FANG have been added to figure 2a.

**Comment:**

L493-4 Data for Olyset and PermaNet3.0 are not shown

**Response:**

The data for olyset and permaNet3.0 were not presented in the figures, as all mosquitoes were dead for PermaNet 3.0 while we had only 3 dead for olyset.

**Comment:**

Things that must be clarified

The intergenic region length : from Fig1B, the CYP6BP5_CYP6P9b intergenic appears to be ~1kb long in the absence of the 4.3 kb element. However, S2 Table refers to ~50 intervals of 1 kb (probably the whole rp1 locus and not the CYP6BP5_CYP6P9b intergenic region). Please clarify.

**Response:**

We agree with the reviewer, Table S3 not S2 is for the entire rp1 locus and shows the calculated Tajima´s D values calculated in 1kb windows. This has been rectified in the supplementary file title of table S3 “Table **S3:** Tajima’s D values across the rp1 locus. The intergenic region between *CYP6P5* and *CYP6P9b* genes is shown in bold at position 8556001 to 8557000.”

**Comment:**

L592-3 The described data are not shown on Table 3 and neither congruent with Fig 4B.

**Response:**

We have added table “ Table S5: Allelic frequencies of the 4.3kb SV among plasmodium infected and non-infected mosquitoes.” to the supplementary which shows the allelic frequencies described in Line 517.

**Comment:**

L361-362: Tajima’s D values are said to be low, in comparison to what ?? And what should be concluded from this observation ?

**Response:**

The low Tajima’s D (D<0) values here refer to the negative values observed in these populations compared to FANG which indicate positive selection or selective sweep in this populations: this line has been rewritten to

Line 363-371:

“This reduction was accompanied by low Tajima’s D values (D<0) in the rp1 locus (including the intergenic region between *CYP6P5* and *CYP6P9b* genes) with negative values observed in Uganda, Ghana and Cameroon compared to FANG (S3 Table). These negative Tajima´s D values indicate positive selection or selective sweep across this locus in these populations.”

**Comment:**

Writing

The Result section dealing with Fig3/Table1-2 describes 5 parallel experimental approaches of essentially the same question , in a lengthy description which takes 5 pages and makes the reader seasick with statistics copy-pasted one by one from the Tables. Because the statistical analysis for these 5 variant experiments is the same, and their take home message is basically also the same, describe the 5 variants of the experiment first, and then state the common conclusions from the statistic tests once and only.

**Response:**

Thanks for pointing this out, we have rewritten this section as suggested and sent the details to the supplementary files.

Line 455-483 And SI results.

**Comment:**

The various pyrethroids/nets used are not appearing in the Discussion. Including their composition in Table 1 or in a Supp Table would help.

L69 explicit LLINs

**Response:**

We have listed examples of LLINs.

**Line 69-72:** All LLINs currently used (PermaNet 2.0, Olyset, Duranet), including the new generation nets (PermaNet 3.0, Olyset plus and Interceptor G2).

**comments:**

L216 Visualizing diversity across the rp1 locus?

**Response:**

This has been corrected.

**Line 217: Visualising nucleotide diversity across the rp1 locus.**

**Comment:**

L267 repeat of line241: to delete

**Response:**

The sentence has been modified.

Line 267-268: PCR products were visualized as described above.

**Comment:**

L295, The phrasing of ‘the 2-ΔΔCT method incorporating PCR efficiency’ is self-contradictory (“The ΔΔCT method incorporating PCR efficiency” would be fine).

**Response:**

We have corrected this

Line 293-296 “the ΔΔCT method incorporating PCR efficiency”

**Comment:**

L334, check syntax

**Response**

This has been corrected.

Line 334-336 “The proportion of each genotype in the alive and dead individuals was determined and then used for the odds ratio calculations. “

**Comment:**

L387, 390-1 check syntax

**Response:**

This has been corrected.

Line 391-394: “The shorter of the two ORFs with 394 amino acids (aas) encodes a zinc finger domain, while the longer domain (906 aas) encodes endonuclease/exonuclease/phosphatase and reverse transcriptase domains in line with other Jockey elements.

**Comment:**

L462-3 fuzzy writing: association to WHAT of the SV ?”

**Response:**

This has been clarified. In SI results, Genotyping of 77 mosquitoes exposed to deltamethrin (35 alive and 42 dead) revealed a strong association (χ2=33.8; P<0.00001) between this structural variant and resistance to deltamethrin.

**Comment:**

L480-1 increase is not significant from 0 to 1 copy.

**Response:**

This has been rectified in SI results supplementary file: “The survival probability increases with two copies of SV+ copies compared to just having one.”

**Comment:**

L547-8 Syntax ??

**Response:**

The sentence has been recorrected. In the supplementary SI results –“ Furthermore, we examined the frequency of the 4.3kb SV in the alpha-cypermethrin 60 minutes alive and 10 minutes dead.

**Comment:**

L632 : this insertion or a nearby mutation.

**Response:**

We have added or nearby mutation in the sentence.

Line 556-558: “Such rapid selection indicates that this insertion or a nearby mutation will likely provide mosquitoes with an essential adaptive advantage.”

**Comment:**

L647 comes back on an hypothesis judged very unlikely on line 622: really no need for this…

**Response:**

We agree with the reviewer and have deleted this sentence.

**Comment:**

L676-7, the 4,3 kb region contains a single transposon only. Since it might contain an enhancer, the distance between CYP6P9a and the 4.3kb insertion should be mentioned.

**Response:**

We have added the distance between CYP6P9a and the 4.3kb insertion as suggested.

Line 600: *CYP6P9a* is found at about 4kb downstream of the 4.3kb structural variant.

**Comment:**

Line701-703, P450s are not mentioned in the NO production nor oxidative stress in the two quoted papers. It could be recalled that the reduction in viral load associated with SV+ allele, although significant, is only modest.

**Response:**

Our intention here was to show a link between the ROS produced by p450s and the possibility these ROS could help modulate the mosquito immune response to plasmodium. We have rephrased this sentence and replaced the references.

**Line 629-633 “**We might speculate that this is linked to 4.3kb SV-driven overexpression of *CYP6P9a/b* since P450s are known to produce reactive oxygen species which can modulate mosquito immunity to *plasmodium* helping to eliminate the parasite. The role of P450 cytochromes during *Plasmodium* invasion remains poorly understood.”

**Comment:**

Figures:
On Fig1C, the 4.3 kb insertion appears to overlap partially the CYP6P9b gene.

**Response:**

Thanks for pointing this out. We have rectified it.

Figure 1C

**Comment:**
Table S3/ Fig S1: if genomic stats were computed on the same intervals (as said L 222-224), why show the output separately rather than stacked on a single Figure ?

**Response:**

Yes, they were computed on the same interval but present different population statistics. Table s3 shows the commuted Tajima`s statistic while figure S1 represents ratios of nucleotide diversity.

**Comment:**

Also, it may help to read the raw numbers of dead/alive mosquitoes on the Fig3 and Fig4B histograms

**Response:**

We have included the raw data for the genotyping experiment in supplementary file 2

**Comment:**
S3Fig is not called in Results.

**Response:**

S3 Fig is referred to in line 431 and L437.

In Tibati, a temporal comparison of the frequency of the 4.3kb structural variant in samples collected in 2018 revealed a 76% SV+ allele frequency and complete fixation of SV+ in 2021 (S3A Fig).

A similar pattern was observed in Mibellon, with the structural variant present at a low allelic frequency (26%) in 2016, increasing to 98% in less than 2 years (2018) and then to 100% in 2020 (S3B Fig).
